# Comparison of four routinely used vitamin D automated immunoassays

**DOI:** 10.5937/jomb0-27531

**Published:** 2021-06-05

**Authors:** Jindra Windrichova, Pavel Broz, Radka Fuchsova, Ondrej Topolcan, Ladislav Pecen, Otto Mayer, Radek Kucera

**Affiliations:** 1 University Hospital Pilsen, Department of Immunochemistry Diagnostics, Czech Republic; 2 University Hospital Pilsen, Institute of Clinical Biochemistry and Hematology, Czech Republic; 3 University Hospital Pilsen, Second Internal Clinic, Czech Republic

**Keywords:** 25(OH)-vitamin D, vitamin D, Unicel, Architect, Cobas, Liaison, method comparison, 25(OH)-vitamin D, vitamin D, "Unicel", "Architect", "Cobas", "Liaison", poređenje metoda

## Abstract

**Background:**

To compare four automated immunoassays for the measurement of 25(OH)-vitamin D (25-OHD) and to assess the impact on the results obtained from a healthy population.

**Methods:**

We analysed 100 serum samples on Unicel DxI 800 (Beckman Coulter), Architect i1000 (Abbott), Cobas e411 (Roche) and Liaison XL (DiaSorin). Passing-Bablok regression and Bland-Altman plots were used for method comparison. In order to categorise the obtained values, results were categorised into the following groups: 0-25 nmol/L, 25-50 nmol/L, 50-75 nmol/L and above 75 nmol/L and compared. The percentage of samples below 75 nmol/L, and below 50 nmol/L was then calculated for every method.

**Results:**

According to paired comparisons, each method differs from others (p<0.0001) except Cobas vs Architect, which do not show a statistically significant difference (p=0.39). The strongest correlation was found between Liaison and Architect (ρ=0.94, p<0.0001). The percentage of samples below the recommended value of 75 nmol/L were: 70% (Architect), 92% (Liaison), 71% (Cobas) and 89% (Unicel). The percentage of samples below the value of 50 nmol/L were: 17% (Architect), 55% (Liaison), 28% (Cobas) and 47% (Unicel).

**Conclusions:**

The observed differences stem from the use of different analytical systems for 25-OHD concentration analysis and can result in different outcomes. The recommended values should be established for each assay in accordance with the data provided by the manufacturer or in the laboratory, in accordance with proper standardisation.

## Abstract (sr)

**Uvod:**

Cilj je bio da se uporede četiri automatizovana imunotesta za merenje 25(OH)-vitamina D (25-OHD) i da se proceni uticaj na rezultate dobijene na uzorku zdrave populacije.

**Metode:**

Analizirali smo 100 uzoraka seruma na analizatorima Unicel DxI 800 (Beckman Coulter), Architect i1000 (Abbott), Cobas e411 (Roche) i Liaison KSL (DiaSorin). Za upoređivanje metoda su korišćene Passing-Bablok-ova regresija i Bland-Altman-ovi plotovi. Da bi se dobijene vrednosti kategorizovale, rezultati su kategorisani u sledeće grupe: 0-25 nmol/L, 25-50 nmol/L, 50-75 nmol/L i iznad 75 nmol/L i posle toga upoređeni. Potom je izračunat procenat uzoraka ispod 75 nmol/L i ispod 50 nmol/L za svaku metodu.

**Rezultati:**

Prema uparenim poređenjima, svaka metoda se razlikuje od ostalih (p <0,0001), osim u slučaju kada se porede rezultati analizatora Cobas i Architect, koji ne pokazuju statistički značajnu razliku (p = 0,39). Najsnažnija korelacija je uočena između Liason i Architect (ρ = 0,94, p <0,0001). Procenat uzoraka ispod preporučene vrednosti od 75 nmol/L je bio: 70% (Architect), 92% (Liaison), 71% (Cobas) i 89% (Unicel). Procenat uzoraka ispod vrednosti od 50 nmol/L je bio: 17% (Architect), 55% (Liaison), 28% (Cobas) i 47% (Unicel).

**Zaključak:**

Uočene razlike potiču od upotrebe različitih analitičkih sistema za analizu koncentracije 25-OHD i mogu da rezultiraju različitim ishodima. Preporučene vrednosti treba da budu utvrđene za svaki test u skladu sa podacima koje je dostavio proizvođač ili u laboratoriji, u skladu sa odgovarajućom standardizacijom.

## Introduction

Vitamin D is involved not only in bone metabolism but also in cardiovascular, neurological and autoimmune diseases, as well as tumorigenesis. Vitamin D concentrations are routinely measured in clinical practice and research. The best marker of vitamin D levels in the body is 25(OH)-vitamin D (25-OHD), present in the highest quantity in the blood. 25-OHD concentrations reflect both the endogenously synthesised and the exogenous form of vitamin D contained in food and supplementation [1]. However, the insufficient comparability of results between the analytical systems in use, as well as general analytical difficulties, lead to uncertainty in defining deficiency in a population. Subsequently, difficulties in interpreting vitamin D values impair the effective use of vitamin D measurements in both routine practice and clinical research.

Vitamin D analysis is a demanding analytical task. The analytical difficulties encountered are related to its lipophilic nature, a strong affinity to vitamin D binding protein (VDBP), the existence of two molecular forms (D2 and D3) and the presence of interfering metabolites (e.g. 24,25-dihydroxy vitamin, C3-epimer of 25-OHD3). Nowadays, 25-OHD immunoassays are required to detect 25-OHD2 and 25-OHD3 in an equimolar way and report a total 25-OHD result [2] [3]. The first analytical method for measurement of 25-OHD concentration was described in the 1970s based on chromatography principles. In 1985, a radioimmunoassay measurement (RIA) was developed based on a specific antibody, becoming the first of its kind to be approved by the Food and Drug Administration (FDA) for clinical diagnostics of vitamin D deficiency. Consequently, methods based on enzymatic detection or chemiluminescent immunoassays (CLIA) were introduced. The progress in tandem mass spectrometry enabled the introduction of a routine LC MS/MS method in 2004 [4].

The Vitamin D Standardisation Program (VDSP) was organised in 2010 by the office of Dietary Supplements of the National Institutes of Health (NIH). It involved the effort of many international organisations to support the standardisation of the 25-OHD measurement in order to improve patient care. It is characterised by five steps but started with the development of a reference sample for vitamin D by the National Institute of Standards and Technology (NIST) in order to enable validation of methods [5] [6]. The material is called Standard Reference Material (SRM) 972, »Vitamin D in Human Serum«, and involves obtaining four blood serum sample pools (Level 1-Level 4) with varying levels of 25-OHD. It possesses certified values for 25-OHD2, 25-OHD3, and 3-epi-25OHD3. The certified concentration values for these analytes are measured by isotope dilution liquid chromatography-tandem mass spectrometry (ID-LC-MS/MS).

The last decade has been characterised by the widespread use of automated immunoanalytical methods for the measurement of 25-OHD concentration. One of the latest immunoassays for 25-OHD analysis to be released was provided by Beckman Coulter; it was introduced in 2014 and approved by the FDA in 2015. The goal of our present study is to compare four automated immunoassays for 25-OHD measurement: 3 CLIA methods and one electrochemiluminescence (ECL) method available in our laboratory; and to compare the differences in the results obtained from a healthy population.

## Methods

### Study population

Participants in our present study, residents of Pilsen, the Czech Republic, underwent the examination as part of the Czech Monica study in 2008. Randomly selected samples were used for comparison of methods. None of the participants had a history of cardiovascular disease, diabetes mellitus, nor were they taking medication for chronic disease. The age (data presented as mean [SD]) of participants was 46 (10.6) years, BMI 26.8 kg/m^2^. 48% of participants were men, 37% of participants were smokers.

### Sample collection

100 serum samples were used for analysis. Samples were collected in the autumn 6^th^–29^th^ October). All blood samples were taken from a peripheral vein using VACUETTE^®^ Z Serum Sep (Greiner Bio-One, Kremsmünster, Austria) tubes. Samples were allowed to clot and were then separated by centrifugation at 1700 g for 10 min. All samples were immediately aliquoted and frozen. Samples were stored at -70°C until the analysis took place. Samples were thawed once, just before measurement.

### Sample analysis

We compared 4 automated methods for 25-OHD measurement accessible in our laboratory, including 3 methods based on CLIA and one based on an ECLprinciple. We performed the 25-OHD assay using the following instruments: Unicel DxI 800 (Beck man Coulter, Brea, CA, USA), presented as »Unicel« in the text, Architect i1000 (Abbott Labo ratories, Libertyville, IL, USA), presented as »Architect«, Cobas e411 (F. Hoffmann – La Roche, Basel, Switzerland), presented as »Cobas«, Liaison XL (Dia Sorin, Saluggia, Italy), presented as »Liaison«.

All instruments are routinely used in the laboratory, and measurements were performed according to the instructions for use as provided by manufacturers and in accordance with good laboratory practice. Our laboratory is a participant in the national external quality control scheme SEKK and the international DEQAS program for 25-OHD measurements.

### Statistical analysis

During the verification procedure of the tested methods, the following basic analytical performance indexes were assessed: Repeatability (intra-assay precision) at 6 serum levels (range 25-91 nmol/L) analysed in hexaplets, intermediate precision (inter-assay precision) using control samples recommended by the manufacturers at 2-3 levels (as defined by the manufacturer) with more than 10 repeated measurements. Bias was calculated using the measurements of DEQAS samples no. 451-455 (characterisation of samples accessible on DEQAS). Values of relative bias were determined in order to calculate the method's specific mean for samples obtained from DEQAS: bias towards the ALTM mean obtained from DEQAS and towards the NIST total 25-OHD2 plus 25-OHD3 value reported in DEQAS.

All presented values are in nmol/L units. Data are presented as median, minimum–maximum, 2.5–97.5 and 5–95 percentile range. Box-plots were constructed for each method from all measured serum values. Wilcoxon signed-rank test (paired test) was used to compare values between methods. Significance was set at P<0.05. Method comparison was further performed using Spearman’s rank correlation and Passing-Bablok regression. Bland-Altman plots were additionally constructed for a better presentation of method comparison.

We categorised the obtained values into the following groups: 0-25 nmol/L, 25-50 nmol/L, 50-75 nmol/L and above 75 nmol/L for a better presentation of differences between analytical systems. The values mentioned above were selected following the recommendations of Endocrine Society [7]. A Chisquare test was used for the comparison of results obtained for each group. To better compare differences, we calculated percentages below 75 nmol/L and below 50 nmol/L for every method. The statistical significance was set at P<0.05.

## Results

**Figure 1:**
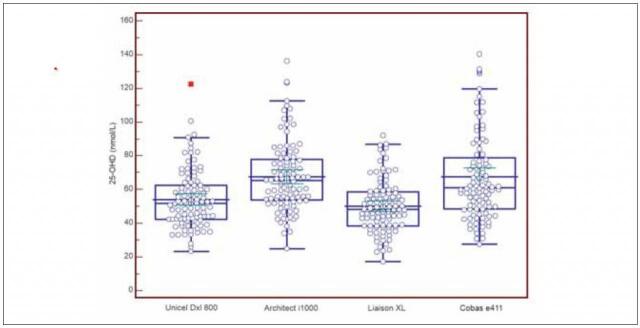
Boxplots of 25-OHD concentrations in 100 samples measured by 4 automated immunoanalytical methods

The descriptive statistics data of 25-OHD as measured by 4 automated immunoanalytical methods in 100 serum samples are listed in Table 1. Median values obtained from the population ranged between 48.1 nmol/L (Liaison) and 65.2 nmol/L (Architect), 2.5 percentiles ranged between 23.5 nmol/L (Liaison) and 35.0 nmol/L (Architect) and 97.5 percentiles between 86.3 nmol/L (Liaison) and 130.5 nmol/L (Cobas). Box-plots of measured concentrations are shown in Figure 1. Variation coefficients of repeatability, intermediate measurement precision and relative bias to DEQAS samples obtained during the verification procedure are listed for all of the 4 immunoanalytical methods in Table 2. Measurement repeatability of studied methods ranges between 5.78% (Unicel) and 3.0% (Liaison) and intermediate precision ranges between 9.83% (Cobas) and 3.29% (Architect).

**Table 1:** Descriptive statistics of 25-OHD values as measured by 4 automated immunoanalytical methods in 100 serum samples taken from a healthy population in the Czech Republic. Percentage of samples bellow 50 nmol/L and 75 nmol/L are included in the table

	Median 1^st^–3^rd^ quartile (min–max) (nmol/L)	2.5–97.5 percentile (nmol/L)	5–95 percentile (nmol/L)	Samples <50 nmol/L (%)	Samples <75 nmol/L (%)
Architect i1000	65.2 53.7–77.8 (24.9–136.2)	35.0–123.3	41.3–108.8	17%	70%
Unicel Dxl800	51.6 42.5–62.4 (23.4–122.5)	27.9–92.5	33.1– 85.8	47%	89%
Liaison XL	48.1 38.6–58.4 (17.0–92.0)	23.5–86.3	26.8–82.7	55%	92%
Cobas e411	61.0 48.6–78.9 (27.6–140.4)	30.9–130.5	35.1–129.4	28%	71%

**Table 2:** Analytical performance of methods – method comparison during verification procedure. ALTM – All-Laboratory Trimmed Mean, DEQAS – Vitamin D External Quality Assessment Scheme, NIST – National Institute of Standards and Technology, 25-OHD2 – 25-hydroxyvitamin D2, 25-OHD3 – 25-hydroxyvitamin D3

	Repeatability (intra-assay precision) (%)	Intermediate precision (inter-assay precision) (%)	% Bias to DEQAS method means	% Bias to DEQAS ALTM	% Bias to DEQAS sample – NIST total 25-OHD2 + 25-OHD3
Architect i1000	2.7	3.29	-5.72	-4.37	1.01
Unicel Dxl 800	6.2	9.42	-1.84	-5.92	-0.57
Liaison XL	2.7	7.63	-15.48	-24.96	-19.67
Cobas e411	5.3	9.83	5.73	4.15	9.48

**Figure 2:**
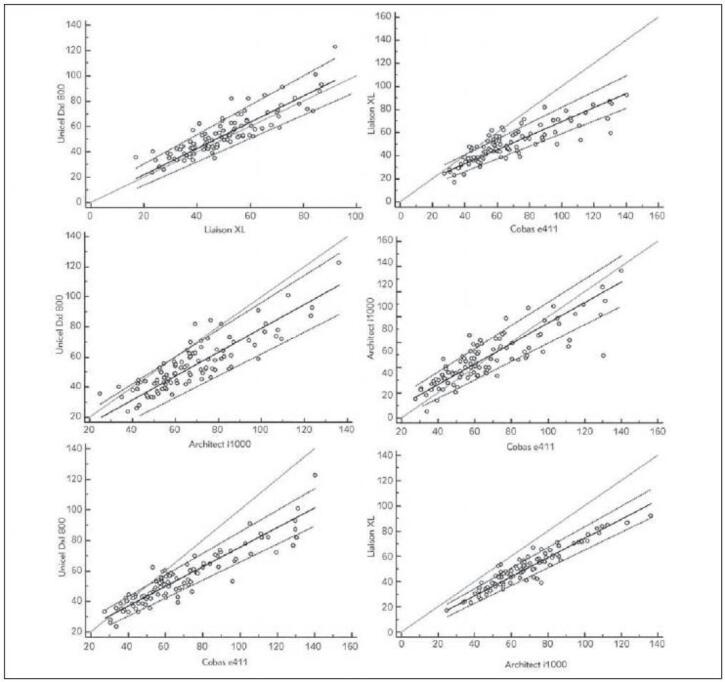
Passing-Bablok regression plots to illustrate differences between methods. Data from Passing-Bablok analysis are stated in Table 3

**Figure 3:**
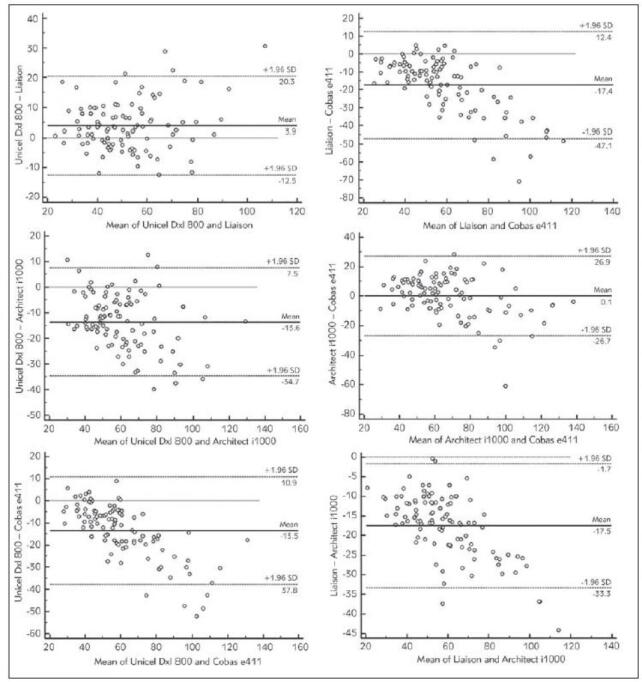
Bland-Altman plots for better illustration of differences between methods

According to paired comparisons of the 4 methods examined, each method differs from the others (p<0.0001). An exception is Cobas vs Architect, which do not show a statistically significant difference (p=0.39). The strongest correlation was found between Liaison and Architect (ρ=0.94, p<0.0001). The second strongest rank correlation was shown between Unicel and Cobas (ρ=0.92, p<0.0001). Surprisingly, methods which do not differ in their pair test (Cobas and Architect) correlate weakly (ρ=0.86, p<0.0001) – this demonstrates that deflections occur, but not systematically in one direction. The conclusions derived from the linear correlation coefficients are identical. Data, including details of correlation analysis and Passing-Bablok regression, are listed in Table 3. Plots of Passing-Bablok regression for the tested methods are presented in Figure 2. Bland-Altman plots are presented in Figure 3 to illustrate the differences between tested methods better.

**Table 3:** Method comparison. Spearman’s rank correlation coefficient, Wilcoxon signed-rank test and Passing-Bablok regression of the studied analytical methods

	Type of statistical analysis			ARCHITECT i1000		Unicel Dxl 800		Liaison XL		Cobas e411	
Architect i1000	Correlation coefficient	p<0.0001				0.851		0.907		0.847	
	Wilcoxon Signed Rank test					p<0.0001		p<0.0001		p=0.39	
	Passing-Bablok	Additive	Slope			1.39	1.24	2.68	1.31	13.21	0.83
Unicel Dxl 800	Correlation Coefficient	p<0.0001		0.851				0.879		0.902	
	Wilcoxon Signed Rank test			p<0.0001				p<0.0001		p<0.0001	
	Passing-Bablok	Additive	Slope	-1.12	0.81			1.92	1.02	10.70	0.66
Liaison XL	Correlation Coefficient	p<0.0001		0.907		0.879				0.841	
	Wilcoxon Signed Rank test			p<0.0001		p<0.0001				p<0.0001	
	Passing-Bablok	Additive	Slope	-2.05	0.77	-1.88	0.98			7.86	0.63
Cobas e411	Correlation Coefficient	p<0.0001		0.847		0.902		0.841			
	Wilcoxon Signed Rank test			p=0.39		p<0.0001		p<0.0001			
	Passing-Bablok	Additive	Slope	-16.0	1.21	-16.31	1.53	-12.51	1.59		

The percentage of samples below the recommended value of 75 nmol/L ranged between 70% (Architect) and 92% (Liaison). The percentage of samples below the value of 50 nmol/L ranged between 17% (Architect) and 55% (Liaison) – values for each method are listed in Table 1. The categorisation of values obtained by each measurement method shows that Cobas and Architect have more optimistic results; a higher percentage of values are evaluated as »normal«, contrary to Unicel and Liaison. The com parison of methods after categorisation (0-25 nmol/L, 25-50 nmol/L, 50-75 nmol/L and values above 75 nmol/L) shows no statistically significant differences in the comparisons of Unicel vs Liaison (p=0.14) and Cobas vs Architect (p=0.11). On the contrary, statistically significant differences were found in the comparison between Unicel and Architect (p<0.0001), Unicel and Cobas (p<0.0001), Liaison and Architect (p<0.0001) and Liaison and Cobas (p<0.0001).

## Discussion

In our present study, we compared four automated immunoassays for 25-OHD measurement accessible in our laboratory: 3 CLIA systems and one ECL system. We focused mainly on the impact of different analytical systems on the clinical classification of patients. The establishment of proper recommended values is important to clinical and laboratory workers alike. Population studies mostly define recommended values based on healthy individuals. Due to our modern lifestyle, the serum values of 25-OHD are generally lower than what is physiologically needed by the human organism. Vitamin D is recognised insufficiency as a risk factor for various diseases by various studies. In our study, we compared the number of individuals selected in subpopulations with concentrations of 25-OHD lower than 75 nmol/L an lower than 50 nmol/L according to the analytical method used. These values were selected because the recommended optimal concentration of 25-OHD is usually higher than 30 ng/mL (75 nmol/L) and 25-OHD values below 20 ng/mL (50 nmol/L) are defined as a deficiency [8]. Percentages of samples categorised into defined groups are summarised in Table 1. Concentrations of 25-OHD were lower than 50 nmol/L in 17-55% of the samples assessed in our study and in 70-92% cases concentrations were lower than 75 nmol/L. Architect and Cobas tend to show more optimistic results in comparison with Liaison and Beckmann. We can agree with Lai et al. [9] in their assessment that the use of different analytical systems for vitamin D measurements might impact clinical decision making.

Given the fact that samples in our study were collected at the beginning of the autumn, we can conclude that a relatively high percentage of the population has vitamin D deficiency. According to Cashman et al. [8] 13.0% of the 55,844 European individuals in their study had serum levels of 25-OHD below 30 nmol/L on average throughout the year. 17.7% of the samples were below 30 nmol/L during the extended winter (October-March), and 8.3% of samples were below the same limit from April to November. According to the definition of vitamin D deficiency as values below 50 nmol/L, the prevalence was 40.4% [8]. It is a well-known fact that a seasonal rhythm of vitamin D exists, but recommended values taking into account seasonal variation are used only in Australia [10]. Recommended values that take seasonal fluctuation into account might be more suitable than a fixed limit in the detection of abnormal concentrations. Moreover, ethnicity, BMI, age and sex should be considered as a source of biological variability while establishing appropriate recommended values [3]. Predictive models reflecting the biological and seasonal variations have been proposed by Vuistiner et al. [11]. Although the seasonal variation for 25-OHD is well described, there is a lack of information on other variabilities. Biological variability data on 25-OHD are not listed in the largest database by Ricos et al. [12], or the EFLM Biological Variation Database [13].

For defining analytical requirements, it is necessary to know the biological variability of an analyte. Viljoen et al. [14] published a study showing that the within-subject variation of 25-OHD was 12.1%, and the between-subject variation was 40.3%. The critical difference was calculated as 38.4%. Objective analytical quality goals have also been established: a minimum achievable performance for the imprecision of ~6% and the desirable analytical bias of ~10% [14]. The performance criteria set by VDSP are a CV of 10% and a bias of 5% [15] [16].

All analytical methods presented in our study are sufficient for the analytical performance of routine use in the clinical laboratory if we consider intra-and inter-assay precision. Regarding bias, only Beckman and Architect are below the limits; the relative percentage bias to DEQAS samples was higher for Cobas (9.48%) and Liaison (-19.67%). Liaison exhibited the highest bias to the DEQAS method means (-15.48%). The analytical performance of Unicel in 25-OHD analysis and its comparison with Liaison was published by Ozcan et al. [17]. They found a correlation with R=0.9498 (intercept 0.528, slope 1.029), and an average bias of 1.2%.

Nowadays, two accuracy-based PT/EQA schemes exist in the world: the accuracy-based vitamin D survey (ABVD) provided by the College of American Pathologists and DEQAS. The DEQAS program started in 1989 and nowadays reports over 1000 participating laboratories [18]. According to a DEQAS review, five out of six fully automated methods had a bias within the VDSP limit in April 2017. However, Abbott-Architect and Siemens-Advia Centaur showed a dependence of bias values on concentration. Additionally, in April 2017, two automated methods had a mean CV below the VDSP threshold (10%). Nevertheless, despite the overall increase in accuracy of 25-OHD assays, caused partly by standardisation procedures, automated ligand binding assays have probably reached their limit [19].

One of the discussed causes for differences between immunoassays is their specificity for D2 and D3 molecules. Serum 25-OHD concentration should be the total of the 25-OHD3 and 25-OHD2 concentrations [15]. Even if the analytical systems tested in our presented study are not equal in their specificity to detect both vitamin D forms, we do not attribute the differences observed between methods to these discrepancies. Generally, only very few samples contain significant levels of 25-OHD2 in Europe, where it is rare for supplements to contain vitamin D2. Food intake in the form of D2 in Central Europe is not expected to be responsible for such differences. Various cross-reactivities of the used immunoassays to other metabolites, e.g. 24,25-(OH)_2_ D3 or 3-epi-25OHD3 can be an additional source of differences in our results [3]. Sample no. 452 from DEQAS measured in our study demonstrated the different performance of Unicel and Cobas when samples include 3-epi-25OHD3. This observation correlates with the cross-reactivity of this metabolite that is described in the tested assays' instructions for use; i.e. 65% for Unicel, 91% for Cobas compared to 2.7% in Architect and 1.3% in Liaison. Not excluding this epimer from the measurement could lead to positive bias and subsequently to errors in clinical decisions when a fixed cut-off point is used to assess vitamin D status; especially in young children, in whom the 3-epimer is present at higher concentration [15]. We assume that the primary source of differences observed between analytical systems in our study can be caused by incomplete extraction from vitamin D binding protein (VDBP) among assays, mostly involving a pH change procedure. Heijboer et al. [20] described the inverse relationship between VDBP and 25-OHD in 4 out of 5 automated 25-OHD assays and the different results in comparison to ID-LC-MS assay. Some authors recommend measuring concentrations of VDBP to evaluate biologically available/free 25-OHD concentration for a better assessment of vitamin D status [21]. However, the influence of VDBP on 25-OHD levels is more complex. Other than the top three common variants, there are more than 120 rare variants, and to our knowledge, their influence has not yet been studied [20] [21]. The differing results could also be caused by matrix substances such as [18]. Finally, another contributing factor can be that the LC-MS/MS reference methods of the manufacturers used for subsequent calibration of immuno assays might not have been harmonised properly.

Ferrari et al. [3] encourage clinical laboratories to adopt assay traceability to the gold SRMP as proposed by VDSP in order to calibrate their new or old measurements. This should be done according to guidelines proposed by VDSP for easy clinical standardisation [5]. Cavalier et al. [22] have shown in their study that the proper method of re-standardisation can improve differences in the results obtained from a healthy population. Nevertheless, problems may remain in specific populations, e.g. pregnant women or dialysis patients [22]. Binkley et al. [23] demonstrate in NHANES III and the KIGGS study on re-standardisation how using non-standardised data can make it impossible to develop valid vitamin D guidelines [23]. According to some authors, the inability to define optimal vitamin D levels despite multiple meta-analyses, including large randomised clinical trials can be partly caused by the use of nonstandardised 25-OHD assays [15]. The VDSP program also introduces a methodology for standardisation that is applicable in the retrospective analysis of existing 25-OHD values measured, e.g. in epidemiological and clinical studies [6].

Our study presents a comparison of four routinely used automated analytical methods for 25-OHD analysis. The strength of the study is in its concurrent use of random population serum samples and »artificial« control samples to evaluate different qualities provided by the automated methods tested. One limitation of the study might be its relatively small number of analysed samples and an absence of patient samples. Another limitation is the lack of comparison with LC-MS method. Additionally, some of the automated assays are not represented in our study, e.g. widely used methods manufactured by Siemens.

Numerous factors discussed above are important while measuring 25-OHD concentrations and establishing recommended values. Each laboratory has to choose its own methodology of measurement and to establish a balance between labour-time, cost-effectiveness, accuracy, specificity and convenience. Based on the results of our study, we would encourage establishing a cut-off value dependent on the specific analytical system in use. This approach, based on manufacturer data, is routinely used in other clinically used assays. A superior approach might be to establish recommended values with cut-offs, alongside proper standardisation of values, in each laboratory.

## Conclusion

The use of different analytical systems for the analysis of 25-OHD concentration can lead to different outcomes. The cut-off variable should be established according to the assay in use and taking into consideration the data provided by the manufacturer. Alternatively, the laboratory should establish its own recommended values in accordance with proper standardisation.

## Author contributions

WJ is responsible for analysis and wrote main parts of the manuscript, PB helped with writing and correcting of the manuscript, FR and MO are responsible for collection and preparation of samples, PL is responsible for statistical analysis, TO is the head of the Department of the Immunochemistry Diagnostics, KR is responsible for the coordination of the whole project.

## Research funding

This study was supported by the Ministry of Health, Czech Republic – conceptual development of research organization (University Hospital in Pilsen – FNPl, 00669806).

## Conflict of interest statement

All the authors declare that they have no conflict of interest in this work.

## List of abbreviations

25-OHD, 25-hydroxyvitamin D; 25-OHD2, 25-hydroxyvitamin D2; 25-OHD3, 25-hydroxyvitamin D3; 3-epi-25OHD3, 3-epi-25-hydroxyvitamin; D3ABVD, accuracy-based vitamin D survey; ALTM, all-laboratory trimmed mean; CLIA, chemiluminescence immunoassay; CV, coefficient of variation; DEQAS, vitamin D external quality assessment scheme ECL, electrochemiluminescence or electrogenerated chemiluminescence; FDA, Food and Drug Administration; NIST, National Institute of Standards and Technology; ODS, Office of Dietary supplements; PT/EQA, proficiency testing/external quality assessment; RIA, radioimmunoassay; SD, standard deviation; SRM, standard reference material; SRMP, standard reference measurement procedure; VDBP, vitamin D binding protein; VDSCP, vitamin D standardisation-certification program; VDSP, vitamin D standardisation program; 24,25-(OH)2D3, 24,25-dihydroxyvitamin D3.
